# Bubble shape instability of acoustic cavitation in molten metal used in ultrasonic casting

**DOI:** 10.1016/j.ultsonch.2024.107064

**Published:** 2024-09-13

**Authors:** Takuya Yamamoto

**Affiliations:** Department of Chemical Engineering, Graduate School of Engineering, Osaka Metropolitan University, 1-1, Gakuen-cho, Naka-ku, Sakai, Osaka 599-8531, Japan

**Keywords:** Acoustic cavitation, Keller-Miksis equation, Shape instability, Aluminum melt, Magnesium melt

## Abstract

In this study, we estimated the equilibrium bubble size of acoustic cavitation in a molten metal, which is basic information in ultrasonic casting. For this, the bubble shape instability of acoustic cavitation in the melt was numerically investigated by solving the Keller–Miksis equation and dynamic equation of the distortion amplitude. The acoustic cavitation bubbles are more stable in aluminum and magnesium melts than in water, and the *parametric instability* mainly determines the bubble stability at 20–160 kHz in molten metals. However, the *afterbounce instability* does not significantly affect the bubble stability in molten metals owing to the small number of bubble oscillations after the first rapid compression, and the distortion amplitude cannot grow significantly after the first compression. The bubbles in the melt become more unstable with an increase in the ultrasonic frequency owing to the corresponding increase in the bubble wall velocity. Through this stability analysis, we can estimate that the stable bubble size in the aluminum or magnesium melt is approximately three or four times larger than that in water at the same ultrasonic pressure amplitude.

## 1 Introduction

When an ultrasound is irradiated into a liquid, acoustic cavitation occurs causing nonlinear bubble oscillations [1], [2]. During these oscillations, the bubble is significantly compressed with a significant increase in the temperature inside the bubble. In addition, the acoustic cavitation bubbles cause shock waves and micro-jet formation [3], [4], [5], [6], [7]. In particular, the water vapor in a bubble is thermally decomposed into radicals, causing many chemical reactions [8], [9]. These phenomena have been used to develop many applications, such as ultrasonic emulsification [10], [11], [12], [13], [14], [15], [16], [17], atomization [18], [19], [20], [21], [22], nano-particle fabrication [23], [24], [25], organic chemical decomposition [26], [27], [28], and ultrasonic casting [29], [30], [31], [32]. In this study, we focused on phenomena that occur during ultrasonic casting.

Ultrasonic casting is an ultrasonic application that has been investigated for a long time [32]. It is expected to be used especially for metals with comparably low-temperature melting points, such as light metals [30], [32]. This is because the lifetime of sonotrodes is significantly short for metals with high-temperature melting points [33]. The solidified microstructures of grains or intermetallic compounds are refined by the ultrasound irradiation [34], [35], [36]. The homogenized size and morphology of these microstructures improve the mechanical characteristics of the cast metal. In addition, grain refiners, which are used to refine the grains of the microstructure, are activated by ultrasound irradiation to remove impurities attached to them [37]. Research has been widely conducted, particularly on aluminum and magnesium alloy melts, to improve the material properties. Attempts have been made to elucidate the phenomena occurring during casting by measuring the sound field distribution in the molten metals [38], [39], [40], acoustic streaming [41], and the direct observation of bubble motion and size [42], [43], [44]. Although many phenomena have been clarified in these studies, some remain unclear because they are difficult to observe. For instance, although the bubble size of acoustic cavitation in an Al-Cu alloy melt was measured using synchrotron observation, the bubble size was measured at a location far from the sonotrode tip owing to the limitation of synchrotron observation [43], [45], and the measured bubble size largely varied in the experiment [46]. In addition, the nanoparticles in the melt increased the bubble size in the melt [47]. Under ultrasound conditions, many bubbles of different sizes exist in the liquid. The cavitation bubble is known to grow owing to the bubble coalescence and rectified diffusion [48], [49]. The sufficiently grown bubbles cannot oscillate when the bubble radius exceeds the resonance radius. Therefore, two types of bubbles exist in a liquid: fine oscillating and large unoscillating. Fine oscillating bubbles emit shock waves and form microjets. Therefore, they are important in ultrasonic casting for the fragmentation of intermetallic compounds and primary crystals. However, active and inactive bubbles cannot be identified separately by the direct observation. Researchers have attempted to measure the time variation of the bubble radius at considerably high speeds, even in the melt. Although bubble motion in a melt with a low-temperature melting point has been measured directly using a high-speed camera [50], measuring the equilibrium bubble size is difficult. Hence, whether the measured bubble size corresponds to the equilibrium bubble size of the melt is unclear. In numerical simulation studies, the equilibrium bubble size in an aluminum alloy melt was set to be almost identical to that in water [51], [52], [53], [54], [55], [56], [57]. In addition to ultrasonic casting in an aluminum melt, ultrasound has been used during the casting of magnesium alloy melt [58], [59], [60]. However, the bubble size of acoustic cavitation in a magnesium alloy melt has not been measured at all, and the equilibrium bubble radius is still unclear.

In the case of acoustic cavitation in water, the equilibrium bubble size has been measured accurately using laser techniques [61], [62], [63], [64]. However, this technique cannot be used for molten metals because of their opacity. In another method, the bubble equilibrium size in water was estimated using stability analysis. Many researchers estimated the stable bubble size by solving both the Rayleigh-Plesset equation and dynamic equation of distortion of spherical harmonics [65], [49], [66], [67], [68], [69], [70], [71], [72], [73] and, also, by solving the Keller–Miksis equation [74], [75], [76], [77], [78], [79] instead of the Rayleigh-Plesset equation. This method was also used to investigate the effects of the surfactants [77], liquid viscosity [74], and ultrasonic frequency [79] in water. In addition, the stabilities of acoustic cavitation bubbles in a glycerin solution [78] and sulfuric mixture [73] were investigated. However, the stability of acoustic bubbles in a molten metal has not been investigated, and the equilibrium bubble radius in the molten metal remains unknown.

The information regarding the bubble stability of acoustic cavitation is significantly important for understanding the phenomena occurring in ultrasonic casting, which is in high demand in the material manufacturing industry. In this study, we used the Keller–Miksis equation and the dynamic equation of distortion to investigate bubble shape instability in aluminum and magnesium melts, which are expected to be used in ultrasonic casting, through stability analysis.

## 2 Numerical analysis

In this study, we solved the Keller–Miksis equation [80] and dynamic equation of distortion of spherical harmonics to simulate the time evolution of the bubble dynamics behavior and small distortions of the bubble shape, respectively. The Keller–Miksis equation was derived from the continuity and Euler equations by including the effect of liquid compressibility, and the dynamic distortion equation was derived from the radial oscillation model equation. The detailed models are presented below.

### 2.1 The Keller-Miksis equation

The bubble radial dynamics were modeled using the following Keller–Miksis equation [1], [80]:

(1) $$\left(1-\frac{\dot{R}}{c_{\infty }}\right)R\ddot{R}+\frac{3}{2}{\dot{R}}^{2}\left(1-\frac{\dot{R}}{c_{\infty }}\right)=\frac{1}{\rho_{L,\infty }}\left(1+\frac{\dot{R}}{c_{\infty }}\right)\left[p_{B}-p_{s}\left(t\right)-p_{0}\right]+\frac{R}{c_{\infty }\rho_{L,\infty }}\frac{dp_{B}}{\mathrm{dt}}$$

where *R* is the bubble radius, *c*_∞_ is the sound speed, *ρ*_L∞_ is the liquid density, *p*_B_ is the liquid pressure at the bubble wall, *p*_s_ is the sinusoidal ultrasonic pressure oscillation, *p*_0_ is the atmosphere pressure, and *t* is time. The liquid pressure at the bubble wall is expressed as

(2) $$p_{B}=p_{g}+p_{v}-\frac{2\sigma }{R}-\frac{4\mu \dot{R}}{R}$$

where *p*_g_ is the pressure inside the bubble, *p*_v_ is the vapor pressure, *σ* is the surface tension, and *μ* is the dynamic viscosity. The pressure inside the bubble is modeled as

(3) $$p_{g}=\left(p_{0}+\frac{2\sigma }{R_{0}}-p_{v}\right){\left(\frac{R_{0}}{R}\right)}^{3\gamma }$$

where γ is the specific heat ratio. The sinusoidal ultrasonic wave is given as

(4) $$p_{s}\left(t\right)=-P_{a}\sin\left(\omega t\right)$$

where *P*_a_ is pressure amplitude, and ω is angular frequency.

In this simulation, to avoid divergence in simulation at high pressure amplitude, the bubble wall velocity ($\dot{R}$) was replaced by the following sound speed in liquid at the bubble wall (*c*_LB_) when the bubble wall velocity exceeded the sound speed (*c*_∞_) [81]. The sound speed in the liquid at the bubble wall was modeled as

(5) $$c_{\mathrm{LB}}=\sqrt{\frac{7.15\left(p_{B}+B\right)}{\rho_{\mathrm{Li}}}}$$

where *B* is the pressure parameter, and *ρ*_Li_ is the liquid density at the bubble wall, which were modeled as in the literature [1], [82]. The sound speed was derived from the modified Tait equation [83].

The physical properties and used parameters are shown in Table 1.

**Table 1:** Physical properties and parameters used in this stability analysis [84], [85].

Properties	Value		Unit
	Aluminum	Magnesium	
Liquid density, *ρ*_L,∞_	2.35 × 10^3^	1.59 × 10^3^	kgm^−3^
External pressure, *P*_0_	1.00 × 10^5^	1.00 × 10^5^	Pa
Liquid sound velocity, *c*_∞_	4.65 × 10^3^	4.00 × 10^3^	ms^−1^
Liquid kinematic viscosity, ν	4.42 × 10^−7^	7.86 × 10^−6^	m^2^s^−1^
Surface tension, *σ*	8.57 × 10^−1^	5.59 × 10^−1^	Nm^−1^
Ratio of specific heat, *γ*	1.40		−
Sound pressure amplitude, *P*_a_	7.00–15.0 × 10^4^		Pa
Equilibrium bubble radius, *R*_0_	5.00–50.0 × 10^−6^		m
Angular frequency, *ω*	20000, 40000, 80000, 160,000 × 2π		rads^−1^

### 2.2 Dynamic equation for small distortion

As introduced by Plesset [65] and Eller *et al*. [49], [66], the fluctuating bubble radius is considered as a linear combination of the bubble radius and distortion of spherical harmonics. The dynamic equation for small distortions of the spherical harmonics can be derived by introducing a fluctuating radius into the dynamic equation of the bubble radius. The dynamic equation for a small distortion is described as

(6) $${\ddot{a}}_{n}+B_{n}\left(t\right){\dot{a}}_{n}-A_{n}\left(t\right)a_{n}=0$$

where *A*_n_ and *B*_n_ are expressed as [70], [71]

(7) $$A_{n}\left(t\right)=\left(n-1\right)\frac{\ddot{R}}{R}-\frac{\beta_{n}\sigma }{\rho R^{3}}-\frac{2\nu \dot{R}}{R^{3}}\left[\left(n-1\right)\left(n+2\right)+2n\left(n+2\right)\left(n-1\right)\frac{\delta }{R}\right]$$

(8) $$B_{n}\left(t\right)=3\frac{\dot{R}}{R}+\frac{2\nu }{R^{2}}\left[\left(n+2\right)\left(2n+1\right)-2n{\left(n+2\right)}^{2}\frac{\delta }{R}\right]$$

where $\beta_{n}$ is modeled as

(9) $$\beta_{n}=\left(n-1\right)\left(n+1\right)\left(n+2\right),$$

where *n* is the degree of spherical harmonics. The boundary layer thickness δ was modeled using Brenner’s model [70], [71] as

(10) $$\delta =\min\left(\sqrt{\frac{\nu }{\omega }},\frac{R}{2n}\right)$$

The boundary layer thickness was modeled as a diffusion length scale, and a cutoff length, *R*/2*n*, was imposed. The time variations of the bubble radius and distortion amplitude were calculated by numerically solving the above Keller–Miksis equation and dynamics equation for small distortions.

After solving these equations, the bubble stability was evaluated using two factors: the magnitude of the maximal eigenvalue of the Floquet transition matrix and the maximal value of the distortion amplitude. The Floquet transition matrix *F*_n_ (*T*) is defined as

(11) $$\left(\begin{array}{c} a_{n}\left(t+T\right)\\ {\dot{a}}_{n}\left(t+T\right) \end{array}\right)=F_{n}\left(t+T\right)\left(\begin{array}{c} a_{n}\left(t\right)\\ {\dot{a}}_{n}\left(t\right) \end{array}\right)$$

where *T* is an ultrasonic cycle period, which is defined by

(12) $$T=\frac{2\pi }{\omega }$$

The distortion amplitude decays when the magnitude of the maximal eigenvalue of the Floquet transition matrix is less than unity. The *parametric instability* was evaluated using this index.

The other factor is the maximal value of the distortion amplitude. During the ultrasonic cycle, the bubble fragments when the distortion amplitude exceeds the bubble radius. In particular, after the first bubble compression, the distortion amplitude begins to increase. This type of instability is called the *afterbounce instability*. The threshold of this instability is calculated as:

(13) $$\max_{\left\{t^{\prime }|t<t^{\prime }<t+T\right\}}\left(\frac{\left|a_{n}\left(t^{\prime }\right)\right|}{R\left(t^{\prime }\right)}\right)\geq 1$$

When the distortion amplitude exceeds the bubble radius, the bubbles are considered unstable.

### 2.3 Numerical methods

The Keller–Miksis equation and distortion equations are discretized using 4th order Runge-Kutta method. The time step for this simulation (Δ*t*) was set to *T*/500,000. At frequencies of 20 and 160 kHz, the time steps were 0.10 and 0.0125 ns, respectively. The initial distortion amplitude was set to 1.0 nm. It should be noted that we previously investigated the effect of the initial distortion amplitude on the stability diagram in our previous study [79] and found that the initial distortion amplitude does not change the stability diagram. Bubble stability was investigated with different bubble radii (5.0–50.0 *μ*m at intervals of 0.10 *μ*m) and pressure amplitudes (7.0 × 10^4^–1.5 × 10^5^ Pa at intervals of 200 Pa). The number of sampled data points in one stability diagram was 450×400 for the bubble radius and pressure amplitude. In our previous study, we validated and verified this program [79].

## 3 Numerical results

### 3.1 Instability in aluminum melt

**Fig. 1:**
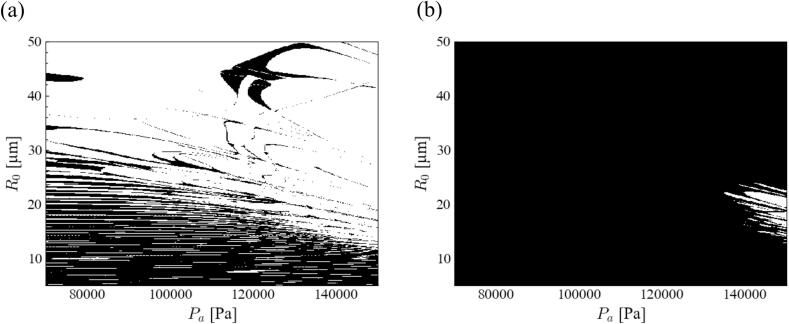
Stability diagram of bubble shape of acoustic cavitation in the aluminum melt at 20 kHz ultrasound when the degree of spherical harmonics is 2: (a) *parametric instability* and (b) *afterbounce instability*.

Fig. 1 shows the stability diagram of the bubble shape of acoustic cavitation in an aluminum melt under a 20 kHz ultrasound. In this graph, the black and white zones indicate the stable and unstable zones for bubble shape instability, respectively. As explained in Section 2.3, the number of data points was 400 and 450 in the horizontal and vertical directions, respectively. The investigated parameter ranges were divided into 400 × 450 squares. When the bubble shape became stable, the square domain was marked in black. Therefore, the spatial resolution of each graph was 400 × 450 pixels, which was sufficiently large. The resolution of the figure might look coarse and unclear. However, this plot of the stable domain originated from the characteristics of the stable zone. The thin stable and unstable band-shaped domains overlapped alternately. Therefore, a locally stable domain was formed, as shown in this figure. The stability diagram of *parametric instability* is completely different from that of *afterbounce instability*. The main stable zone of the *parametric instability* is located at the bottom-left corner of Fig. 1 (a). This implies a spherical bubble shape corresponding to a small pressure amplitude of ultrasound and small bubble radius. Meanwhile, the *afterbounce instability* was hard to occur in this parameter range. Therefore, the bubble became aspherical owing to *parametric instability*. *Afterbounce instability* does not occur easily in any of the other parameters. Hence, we focused only on *parametric instability*.

**Fig. 2:**
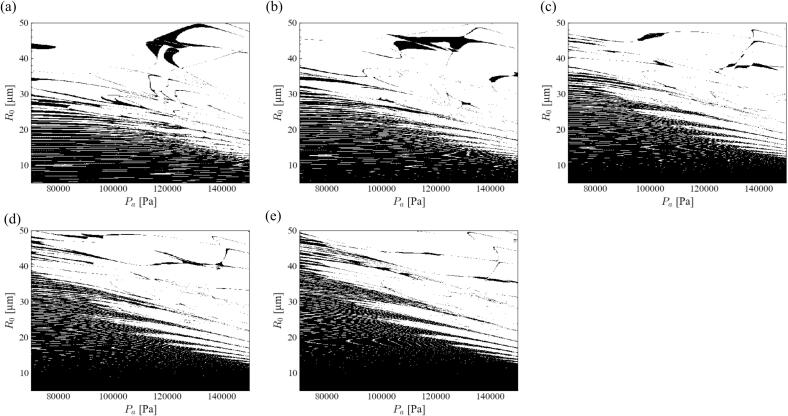
Stability diagram of *parametric instability* in the aluminum melt at 20 kHz ultrasound with different degree of spherical harmonics, *n* = (a) 2, (b) 3, (c) 4, (d) 5, and (e) 6.

Next, we evaluated the influence of the spherical harmonic degree on the stability diagram. Fig. 2 shows the stability diagram of the *parametric instability* in an aluminum melt at 20 kHz ultrasound with different degrees of spherical harmonics. Similar to the acoustic cavitation in water [71], [79], the most unstable mode was *n* = 2. The stable zone expanded slightly with an increase in the degree of spherical harmonics, although a sharp stable band-shaped domain was formed near the boundary between the stable and unstable zones. For example, the boundary at a pressure amplitude of 70000 Pa was located at approximately 30, 35, and 50 *μ*m for *n* = 2, 4, and 6, respectively. Similar to acoustic cavitation in water, Mathieu toughs are formed near the border of stable and unstable zones. Notably, the tough length was much longer, and the tough shape was much sharper in the aluminum melt than in water. In this section, we have focused on the most unstable mode (*n* = 2).

**Fig. 3:**
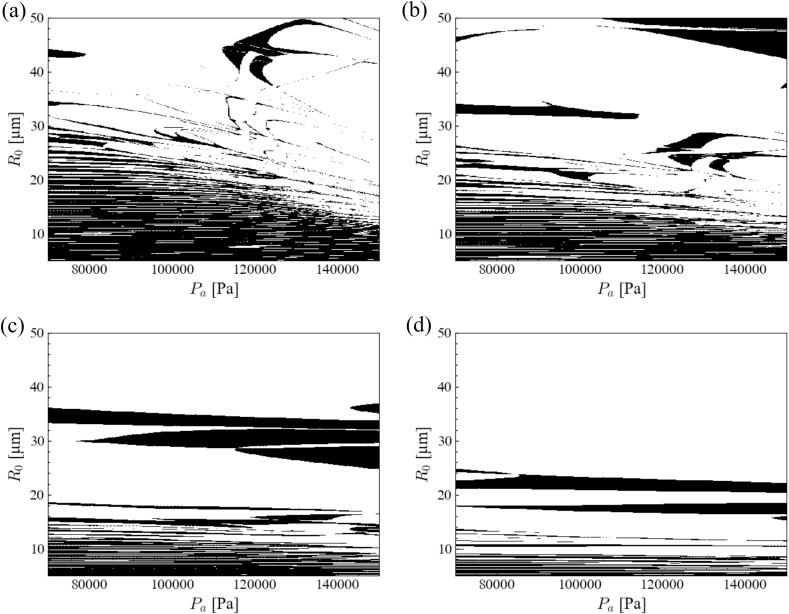
Stability diagram of *parametric instability* in the aluminum melt ultrasound with *n* = 2 degree with different ultrasonic frequencies: (a) 20, (b) 40, (c) 80, and (d) 160 kHz.

The influence of ultrasonic frequency on bubble shape instability was investigated for an aluminum melt. Fig. 3 shows the stability diagram of the *parametric instability* in the aluminum melt ultrasound with *n* = 2 at different ultrasonic frequencies. Similar to *parametric instability* in water, the stable zone in the stability diagram shrank with an increase in ultrasonic frequency. Surprisingly, the stable zone aligned horizontally at higher ultrasonic frequencies. This result indicates that the bubble instability is not affected by the pressure amplitude, but by the bubble radius. When the ultrasonic frequency increased, the bubbles became unstable, and the slope of the border between the stable and unstable zones became gentle. This gentle slope was more remarkable in the case of the aluminum melt, causing a horizontally aligned stable zone.

### 3.2 Instability in magnesium melt

**Fig. 4:**
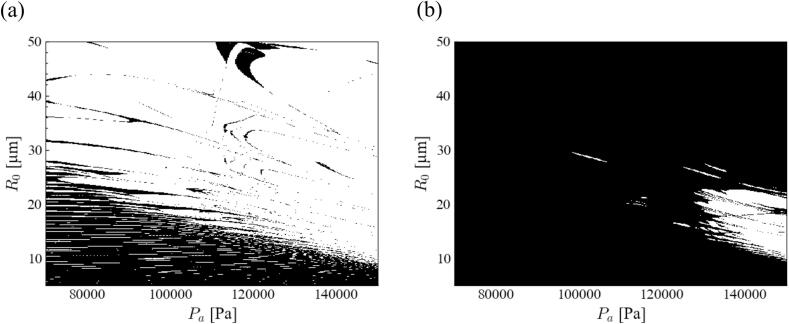
Stability diagram of bubble shape of acoustic cavitation in the magnesium melt at 20 kHz ultrasound when the degree of spherical harmonics is 2: (a) *parametric instability* and (b) *afterbounce instability*.

Fig. 4 shows the stability diagram of the bubble shape of the acoustic cavitation in the magnesium melt at 20 kHz ultrasound when the degree of spherical harmonics was 2. The tendency of the unstable zone in the magnesium melt was the same as that in the aluminum melt. The stable zone of the *parametric instability* for the magnesium melt is completely different from that of the *afterbounce instability*. The stable zone expands widely in the case of *afterbounce instability*. Hence, the most unstable mode is determined by the *parametric instability*. Hereafter, we focused only on the *parametric instability*. Smaller bubbles and pressure amplitudes were required to maintain spherical bubbles. Fig. 1, Fig. 4 were compared, and the stable zone was found narrower in the case of the magnesium melt for *parametric instability*. Hence, the acoustic cavitation bubbles in the magnesium melt are more unstable than those in the aluminum melt.

**Fig. 5:**
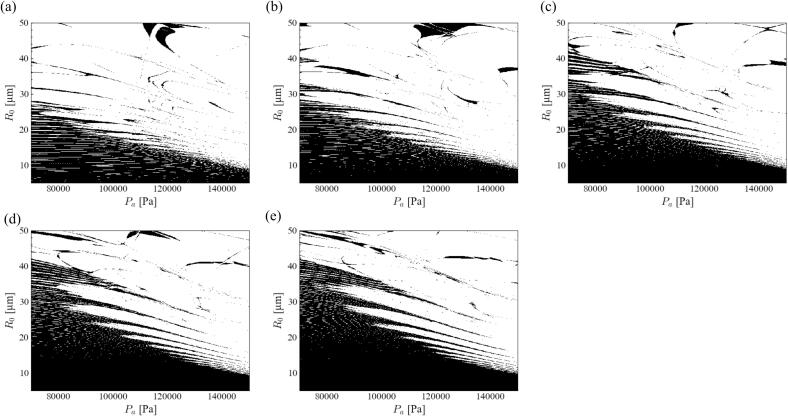
Stability diagram of *parametric instability* in the magnesium melt at 20 kHz ultrasound with different degree of spherical harmonics, *n* = (a) 2, (b) 3, (c) 4, (d) 5, and (e) 6.

Next, we investigated the influence of the spherical harmonic degree on the stability diagram. Fig. 5 shows a stability diagram of the *parametric instability* in the magnesium melt at 20 kHz ultrasound with different degrees of spherical harmonics. Similar to aluminum melt, as shown in Fig. 2, the stable zone expanded with an increase in the degree of the spherical harmonics. The most unstable mode was *n* = 2. Therefore, we focused on the *parametric instability* with *n* = 2.

**Fig. 6:**
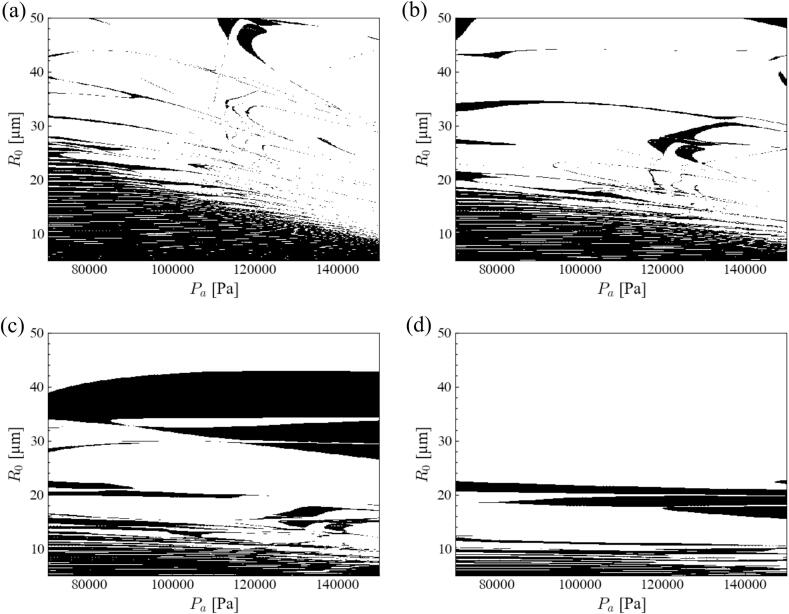
Stability diagram of *parametric instability* in the magnesium melt ultrasound with *n* = 2 with different ultrasonic frequencies: (a) 20, (b) 40, (c) 80, and (d) 160 kHz.

Fig. 6 shows the stability diagram of the *parametric instability* in the magnesium melt ultrasound with *n* = 2 at different ultrasonic frequencies. The tendency of the stable zone in the magnesium melt was the same as that in the aluminum melt. When the ultrasonic frequency increased, the stable zone shrank, and the bubble became unstable. Similar to aluminum melt, the stable zone became horizontally aligned at higher ultrasonic frequencies. In the next section, the mechanism of the above-mentioned phenomena is discussed, and the bubble stability in different liquids is compared.

### 3.3 Discussion

Here, the generation mechanism of bubble shape instability is discussed separately, and bubble instabilities are compared in different liquids.

#### 3.3.1 Generation mechanism of bubble shape instability

In this subsection, the bubble shape instability is discussed in terms of the following three points:

• The reason for bubble instability with an increase in ultrasonic frequency.
• The reason for the difficulty of *afterbounce instability* occurrence in molten metals, although the *afterbounce instability* is most unstable mode in water at 20 kHz [79].
• The reason for the local stable zone to appear at high pressure amplitudes and large bubble radii.

The phenomenon was investigated in our previous study [79]. The moving velocity and acceleration of bubble wall increase with ultrasonic frequency. Hence, the distortion amplitude increases significantly, and the bubbles become unstable at higher ultrasonic frequencies. For further details, the readers are requested to refer to our previous study [79].

In the previous study, *parametric* and *afterbounce instability* occurred simultaneously at 20 kHz in water [79], and the *parametric instability* became dominant at higher ultrasonic frequencies. Meanwhile, *afterbounce instability* did not occur in molten metals, even at low ultrasonic frequencies, as shown in Fig. 3, Fig. 6. Second, we have explained the reason for difficulty of *afterbounce instability* occurrence in molten metal at 20 kHz below.

**Fig. 7:**
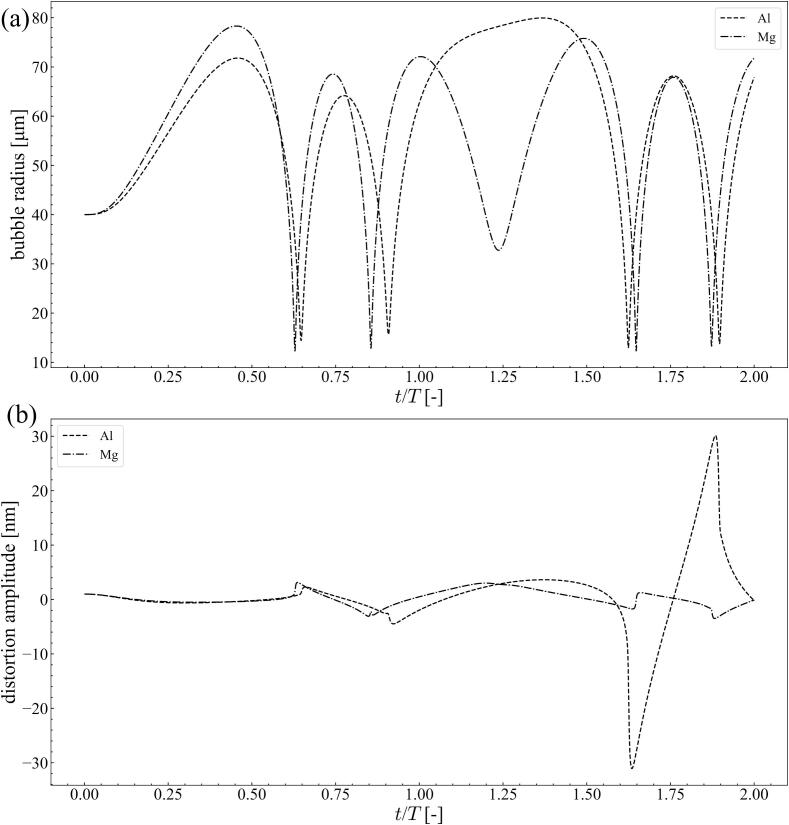
Time variation of (a) bubble radius and (b) distortion amplitude with *n* = 2 at 20 kHz ultrasound with the pressure amplitude of 1.1 × 10^5^ Pa; the bubble radius is 40 *μ*m in the aluminum and the magnesium melts.

**Fig. 8:**
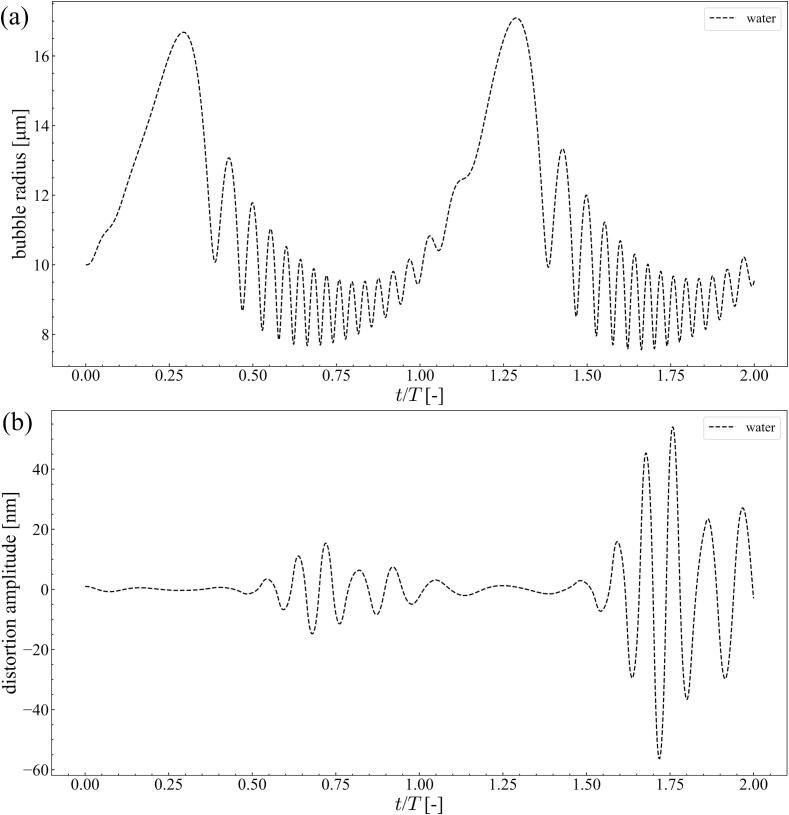
Time variation of (a) bubble radius and (b) distortion amplitude with *n* = 2 at 20 kHz ultrasound with the pressure amplitude of 9.0 × 10^4^ Pa; the bubble radius is 10 *μ*m in water.

Fig. 7 shows the time variation of the bubble radius and distortion amplitude with *n* = 2 at 20 kHz ultrasound with a pressure amplitude of 1.1 × 10^5^ Pa. The bubble radius was 40 *μ*m in the aluminum and magnesium melt. The distortion amplitude increased after the first collapse of the bubble. After collapse, the bubble was bound one or two times before the start of the next ultrasonic cycle. Compared to the bubble oscillations in water at 20 kHz, the number of bounces after the first collapse was small. For reference, the time variations of the bubble radius and distortion amplitude in water are shown in Fig. 8. In this simulation, the pressure amplitude was 9.0 × 10^4^ Pa and the bubble radius was 10 *μ*m. Contrary to the molten metals, small oscillations with high frequency were observed in water after the first collapse of the bubble. During these small bubble oscillations, the distortion amplitude increased significantly and *afterbounce instability* occurred. However, the number of small oscillations after the first collapse is small in the molten metal. Therefore, *afterbounce instability* occurrence is difficult in the molten metal. Conversely, the distortion amplitude increased with the number of ultrasonic cycles in the molten metal, as shown in Fig. 7(a). Therefore, *parametric instability* can occur in molten metals.

**Fig. 9:**
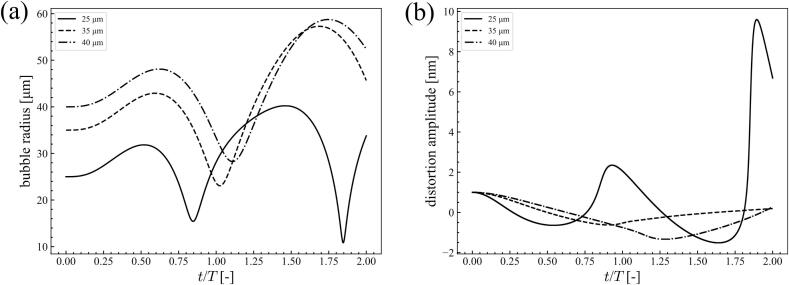
Time variation of (a) bubble radius and (b) distortion amplitude with *n* = 2 at 80 kHz ultrasound with the pressure amplitude of 8.0 × 10^4^ Pa; the bubble radii are 25, 35 and 40 *μ*m in the aluminum melt.

Third, we have discussed the reason for a local stable zone to appear at high pressure amplitudes and large bubble radii below. This phenomenon can be simulated by direct numerical simulations using the volume of the fluid method [86]. Here, we focused on the stability diagram of the aluminum melt under 80 kHz ultrasound, as shown in Fig. 3(c). A long and wide stable domain was observed at approximately *R*_0_ = 30–40 *μ*m. Therefore, the time variations of the bubble radius and distortion amplitude were evaluated for the equilibrium radii *R*_0_ = 25, 35, and 40 *μ*m at a pressure amplitude of 8.0 × 10^4^ Pa. The bubbles were unstable at *R*_0_ = 25 and 40 *μ*m but stable at *R*_0_ = 35 *μ*m. Fig. 9 shows the time variation of the (a) bubble radius and (b) distortion amplitude with *n* = 2 at 80 kHz ultrasound with a pressure amplitude of 8.0 × 10^4^ Pa for the bubble radii of 25, 35, and 40 *μ*m in the aluminum melt. The time at which the bubble was the most compressed shifted later as the bubble size increased. Accordingly, the periods of the bubble and ultrasonic oscillations did not match. Owing to this mismatch, the distortion amplitude did not increase, especially at approximately *R*_0_ = 35 *μ*m.

#### 3.3.2 Comparison of bubble instability between different liquids

**Fig. 10:**
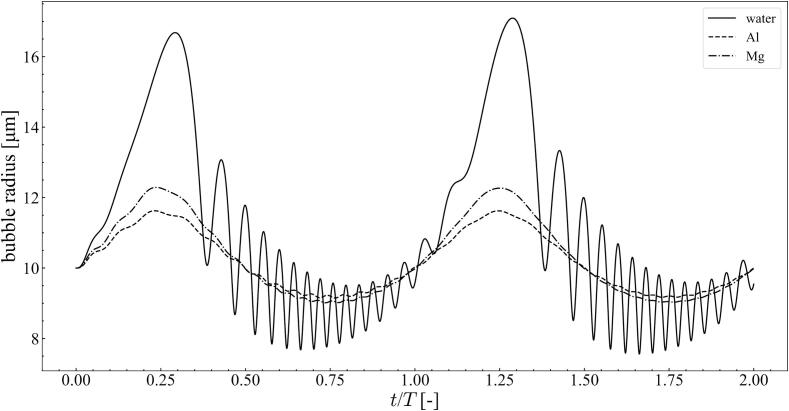
Time variation of bubble radius with different liquids at 20 kHz with the pressure amplitude of 1.0 × 10^5^ Pa, when the bubble radius is 10 *μ*m.

As shown in Fig. 3, Fig. 6, the stable zone in the aluminum melt was slightly larger than that in the magnesium melt. These results were compared with the stability diagram of water reported in the literature [70], [71], [72], [74], [77], [79], [87]: the stable zone was much wider, and the bubbles were more stable in aluminum and magnesium melts. For example, when the ultrasonic frequency was 20 kHz and the pressure amplitude was 1.0 × 10^5^ Pa, the bubbles became unstable at ∼6 *μ*m in water and at ∼20–25 *μ*m in the aluminum and magnesium melts. These results indicate that the bubble radius of acoustic cavitation was larger in aluminum and magnesium melts than in water. This result is in good quantitative agreement with experimentally observed bubbles [43]. Fig. 10 shows the time variation of the bubble radius (=10 *μ*m) with different liquids at 20 kHz with a pressure amplitude of 1.0 × 10^5^ Pa. The bubble oscillation amplitude was considerable small in the case of molten metals. This is because the surface tension of molten metals is one order of magnitude higher than that of water. In addition, the surface tension of the aluminum melt is higher than that of the magnesium melt as shown in Table 1. Therefore, the acoustic cavitation bubbles are more stable in the aluminum melt. The distortion cannot grow owing to the small bubble oscillations at small pressure amplitudes and bubbles in the case of molten metals. The descending order of the bubble oscillation amplitude, as shown in this figure, was the same as that of the bubble instability.

The bubble size and stability were compared with the experimental results [43]. According to the in-situ observation of the Al-Cu alloy system at 30 kHz ultrasound, the bubble modal radius in the aluminum alloy melt was ∼10–20 *μ*m, which was much larger than that in water. The measured bubble modal radius corresponded to the simulated radius when the pressure amplitude was ∼1.0 × 10^5^ Pa. From these results, we can conclude that the stability analysis predicted the stable bubble diameter. The measured bubble size was slightly larger than those in other studies [46], [47]. For example, the most frequent radii are ∼30–50 *μ*m [46] and ∼25 *μ*m [47] in Al-Cu alloy melts. As shown in the stability diagrams, the bubble stability was largely affected by the pressure amplitude, and inactive bubbles were counted in the experiments. Therefore, the variation in the measured values was not significant in this range. No experimentally measured values are available for magnesium alloy melts. However, the bubble size in a magnesium melt can be predicted to be almost the same or slightly smaller than that in an aluminum melt. This information is helpful for understanding the phenomena occurring in ultrasonic casting of magnesium melts.

Previously, the radial dynamics of cavitation bubbles have been solved in aluminum alloy melts [51], [52], [53], [54], [55], [56], [57], [88] and magnesium alloy melts [89], [90]. To investigate ultrasonic processing in aluminum alloy melts, many researchers set the equilibrium bubble radius to 5 *μ*m [51], [52], [53], [54], [55], [56], [57] and to 4.5 *μ*m in magnesium ultrasonic casting [89], [90]. These values were similar to the equilibrium bubble size of water. However, as found in this study, the equilibrium bubble size was much larger than these values. The aforementioned studies used a smaller equilibrium bubble size. So, the influence of the bubble size on the phenomena occurring in ultrasonic casting should be carefully investigated in the future study.

## 4 Conclusion

In this study, a linear stability analysis was conducted to investigate the bubble shape stability and stable bubble size in molten aluminum and magnesium, which are expected to be used in ultrasonic casting applications. The main findings of this study are summarized as follows.

• Acoustic cavitation bubbles in aluminum or magnesium melts are more stable than those in water at the same pressure amplitude. This is because the larger surface tension in the molten metal suppresses bubble oscillation, leading to smaller distortion growth.
• The acoustic cavitation bubble in the aluminum melt is slightly more stable than that in the magnesium melt. This is because the surface tension of the aluminum melt is larger than that of magnesium.
• *Afterbounce instability* does not occur in aluminum and magnesium melts, although it can occur in water at 20 kHz. This is because the number of small bubble oscillations after the first collapse is smaller in the molten metal than in water.
• An acoustic cavitation bubble in an aluminum or magnesium melt is destabilized with an increase in the ultrasonic frequency. This is because the moving velocity and acceleration of the bubble wall increase with an increase in the ultrasonic frequency.
• Stable zones in the stability diagrams of aluminum and magnesium melts exist even with a larger bubble radius. This is because the periods of bubble oscillations and ultrasonic oscillations do not match, leading to less distortion growth.

In a previous study on the ultrasonic casting of aluminum using numerical simulations, the equilibrium radius was set to be much smaller than that derived in this study. Previous studies used the equilibrium radius of water instead of that of a molten metal. In future studies, researchers should reconsider the influence of the equilibrium bubble radius on the numerical results of ultrasonic casting of molten metals.

## CRediT authorship contribution statement

**Takuya Yamamoto:** Writing – review & editing, Writing – original draft, Visualization, Validation, Software, Methodology, Investigation, Funding acquisition, Formal analysis, Data curation, Conceptualization.

## Declaration of competing interest

The authors declare the following financial interests/personal relationships which may be considered as potential competing interests: Takuya Yamamoto reports financial support was provided by Japan Science and Technology Agency. If there are other authors, they declare that they have no known competing financial interests or personal relationships that could have appeared to influence the work reported in this paper.
